# Association between medication-related adverse events and non-elective readmission in acute ischemic stroke

**DOI:** 10.1186/s12883-018-1195-0

**Published:** 2018-11-19

**Authors:** James A. G. Crispo, Dylan P. Thibault, Yannick Fortin, Daniel Krewski, Allison W. Willis

**Affiliations:** 1 Department of Neurology, University of Pennsylvania Perelman School of Medicine, Blockley Hall, 423 Guardian Drive, Office 811, Philadelphia, PA 19104 USA; 2 Department of Biostatistics, Epidemiology and Informatics, University of Pennsylvania Perelman School of Medicine, Blockley Hall, 423 Guardian Drive, Office 811, Philadelphia, PA 19104 USA; 3 McLaughlin Centre for Population Health Risk Assessment, University of Ottawa, 600 Peter Morand Crescent, Room 216A, Ottawa, ON K1G 5Z3 Canada

**Keywords:** Acute ischemic stroke, Readmission, Medication-related adverse events, Nationwide readmission database

## Abstract

**Background:**

There is limited data on the effects of medication-related adverse events occurring during inpatient stays for stroke. The objectives of our study were to characterize reasons for acute readmission after acute ischemic stroke (AIS) and determine if medication-related adverse events occuring during AIS hospitalization were associated with 30-day readmission. Secondary objectives examined whether demographic, clinical, and hospital characterisitcs were associated with post-AIS readmission.

**Methods:**

We used the Nationwide Readmission Database to identify index AIS hospitalizations in the United States between January and November 2014. Inpatient records were screened for diagnostic and external causes of injury codes indicative of medication-related adverse events, including adverse effects of prescribed drugs, unintentional overdosing, and medication errors. Nationally representative estimates of AIS hospitalizations, medication-related adverse events, and acute non-elective readmissions were computed using survey weighting methods. Adjusted odds of readmission for medication-related adverse events and select characteristics were estimated using unconditional logistic regression.

**Results:**

We identified 439,682 individuals who were hospitalized with AIS, 4.7% of whom experienced a medication-related adverse event. Overall, 10.7% of hospitalized individuals with AIS were readmitted within 30 days of discharge. Reasons for readmission were consistent with those observed among older adults. Inpatients who experienced medication-related adverse events had significantly greater odds of being readmitted within 30 days (adjusted odds ratio (AOR): 1.22; 95% CI: 1.14–1.30). Medication-related adverse events were associated with readmission for non-AIS conditions (AOR, 1.26; 95% CI: 1.17–1.35), but not with readmission for AIS (AOR, 0.91; 95% CI: 0.75–1.10). Several factors, including but not limited to being younger than 40 years (AOR, 1.12; 95% CI: 1.00–1.26), Medicare insurance coverage (AOR, 1.33; 95% CI: 1.26–1.40), length of stay greater than 1 week (AOR, 1.38; 95% CI: 1.33–1.42), having 7 or more comorbidites (AOR, 2.20; 95% CI: 2.08–2.34), and receiving care at a for-profit hospital (AOR, 1.20; 95% CI: 1.12–1.29), were identified as being associated with all-cause 30-day readmission.

**Conclusions:**

In this nationally representative sample of AIS hospitalizations, medication-related adverse events were positively associated with 30-day readmission for non-AIS causes. Future studies are necessary to determine whether medication-related adverse events and readmissions in AIS are avoidable.

## Background

Despite improvements in population health contributing to decreasing stroke incidence, mortality, and age-adjusted hospitalization rates in the United States (US) over the last decade [1–3], stroke remains a leading cause of death, hospitalization, and healthcare expenditure [1, 4]. Estimates suggest that approximately 795,000 individuals experience a new or recurrent stroke each year in the US [1], and that readmissions following hospitalizations for stroke are relatively common and often occur for potentially avoidable causes, including urinary tract infections, uncontrolled diabetes, and pneumonia [4–6].

Hospital readmission is considered to be a useful indicator of quality of healthcare services [7–9], which has prompted some governments and insurers to implement hospital reimbursement schedules that are dependent on short-term hospital readmission rates [10, 11]. In effort to better understand factors contributing to readmission after stroke and identify populations at greatest risk of re-hospitalization, a number of studies have examined whether patient and care setting factors are associated with post-stroke readmission [5, 12–15]. Differences in study design, exposure and outcome defintions, and statisitical analyses across studies have led to inconsistencies in reported results [8]. Nonetheless, length of index admission [6, 16, 17], discharge disposition [5], and stroke severity [13, 18] have been identified as consistent predictors of post-stroke readmission and may be essential measures in the development of risk-standardized readmission models for stroke.

Previously, a large multi-center study of stroke readmission in Australia found that experiencing an adverse event or severe complication, such as recurrent stroke, penumonia, urinary tract infection, or fall, during hospitalization was significantly associated with readmission within 28 days (adjusted odds ratio (AOR), 2.81; 95% confidence interval (CI): 1.55–5.12) [19]. Based on these findings, it is important to examine whether a similar relationship exists in the US, and whether the reported association between inpatient adverse events and acute readmission extends to medication-related adverse events. To enhance our understanding of medication-related adverse events among stroke inpatients, we examined acute ischemic stroke (AIS) hospitalizations and readmissions using the US Healthcare Cost and Utilization Project’s (HCUP) 2014 Nationwide Readmissions Database (NRD). Our primary objectives were to characterize reasons for readmission within 30 days of discharge and determine if medication-related adverse events occuring during AIS hospitalization were associated with acute readmission. Our secondary objectives were to examine whether demographic, clinical, and hospital characteristics were associated with post-AIS readmission.

## Methods

### Data source

Using the 2014 NRD, we performed a cross-sectional analysis of hospitalizations for AIS and associated readmissions. Sponsored by the Agency for Healthcare Research and Quality, the NRD is a family of databases developed as part of the HCUP to support national readmission analyses for all payer classes in the US, including the uninsured. The NRD contains detailed demographic (including age, sex, and health insurance status), clinical (such as diagnoses, procedures, and length of stay), and hospital data (size, location, and teaching status) for approximately 35 million annual discharges. Persons admitted to NRD contributing hospitals may be longitudinally followed within calendar years, but not across years.

### Study Population

A validated algorithm for stroke classification was employed to identify all 2014 discharges where a diagnosis of AIS was recorded [20], hereinafter referred to as index AIS hospitalizations. The utilized algorithm was specifically developed for the detection and classification of stroke (AIS, intracerebral hemorrhage, and subarachnoid hemorrhage) using administrative claims data from the US, with a reported sensitivity of 86% and specificity of 95% for AIS discrimination [20]. We identified index AIS hospitalizations by querying all diagnostic fields for documentation of any of the following International Classification of Diseases, Ninth Revision (ICD-9) codes: 433.× 1, 434.× 1, and 436. Index AIS hospitalizations with documented diagnoses of traumatic brain injury (ICD-9 codes: 800.xx-804.xx, 850–854.xx) or primary procedure indicative of rehabilitation care (ICD-9 code: V57) were excluded from the study population. Hospitalizations with undocumented length of stay and encounters where the patient died in-hospital were also excluded. Since source state data for the NRD database employ different patient identifiers, it is not possible to track encounters for the same individual across state boundaries. Therefore, index AIS hospitalizations were restricted to discharges where individuals were a resident of the same state as the hospital where they received care. To ensure the availability of 30-day readmissions data, December discharges of index AIS hospitalizations were also excluded.

### Demographics, comorbidities, and care settings

Personal data extracted from index AIS hospitalizations included age, sex, health insurance status, length of stay, and discharge disposition. Comorbidities documented during the inpatient stay were assessed according to enhanced ICD-9 coding algorithms for Elixhauser comorbidities [21]. A single comorbidity score per encounter was computed as the sum of distinct comorbidities recorded during the hospitalization. Care setting characterisitcs of interest from index AIS hospitalizations included hospital size, geographic location, and teaching status.

### Medication-related adverse events

Medication-related adverse events occurring during the index AIS hospitalization were identified using ICD-9 and External Causes of Injury codes (E codes) indicative of adverse drug events and medication errors [22]. Documentation of one or more of the following ICD-9 or E codes within index AIS hospitalization records served to indicate the occurrence of a medication-related adverse event: 357.6 (neuropathy due to drugs); 692.3 (contact dermatitis due to drugs and medicines in contact with skin); 693.0 (dermatitis due to drugs or medicines taken internally); 960.0–964.9, or 965.02–969.5, or 969.8–979.9 (poisoning by drugs, medicinal and biological substances); E850.1–E858.9 (accidental poisoning by drugs, medicinal substances, and biological substances); and E930.0–E934.9, or E935.1–E949.9 (drugs, medicinal, and biological substances causing adverse effects in therapeutic use). Index AIS hospitalizations with adverse drug events associated with illicit drug use, intentional harm, or poisonings unknown to be accidental were excluded from our analyses: 965.00 (opium poisoning); 965.01 (heroin poisoning); 969.6 (psychodysleptic poisoning); E850.0 (accidental poisoning by heroin); E854.1 (accidental poisoning by hallucinogens); E854.2 (accidental poisoning by psychostimulants); E935.0 (adverse effects of heroin); E939.6 (adverse effects of hallucinogens); E939.7 (adverse effects of psychostimulants); E950.0–E950.9 (suicide and self-inflicted poisoning); E962.0–E962.9 (assault by poisoning); and E980.0–E980.9 (poisoning undetermined to be accidental).

### Readmissions

Same state readmissions within 30 days of index AIS hospitalization discharge were coded as the first readmission within 30 days. In instances where individuals were readmitted on multiple occasions within 30 days, only the encounter with the earliest time to readmission was retained for our analyses. Of these readmissions, elective readmissions were excluded. Time to readmission was calculated as the number of days between index AIS hospitalization discharge date and the date of first readmission. Readmissions where AIS was diagnosed according to the validated algorithm for stroke classifications were deemed to be for AIS. We summarized the principal causes of readmission (first diagnostic position) using HCUP’s single-level Clinical Classifications Software [23], which aggregates ICD-9 diagnosed illnesses and conditions into 285 mutually exclusive and clinically meaningful categories.

### Statistical analyses

Survey weighting methods that accounted for the NRD’s sampling design were used to generate nationally representative estimates of all reported values, including the number of index AIS hospitalizations, the characteristics of these hospitalizations, and subsequent readmissions [24, 25]. Descriptive statistics were used to report individual, cormorbidity, and care setting characteristics for index AIS hospitalizations as a whole and by 30-day readmission status.

The chi-square test was used to make statistical comparisons between populations readmitted and not readmitted within 30 days of index AIS hospitalization discharge.

We examined differences in time to readmission by medication-related adverse event status within quartiles. We created population quartiles for each category of readmission. Applying the survey weights, we then compared proportions across medication-related adverse event status and population quartiles for time to readmission using the Wald chi-square test, which is based on the difference between observed and expected weighted cell frequencies. This approach allowed us to test for independence of medication-related adverse event status and time to readmission quartiles, while taking into account the NRD’s complex survey design.

To examine the association between medication-related adverse events and readmission within 30 days of index AIS hospitalization discharge, we constructed weighted unconditional logistic regression models to estimate adjusted odds of readmission for those experiencing an adverse drug event during their hospitalization (compared to no adverse drug event). All models accounted for the survey design of the NRD and utilized the stratums and clustering of patients and hospitals to generate accurate variance estimates. Secondary analyses used similar models to assess associations between sociodemogaphic factors and readmission within 30 days of index AIS hospitalization discharge. Adjusted models included characterisitics that were hypothesized a priori to potentially confound modelled relationships. All analyses were completed using SAS v9.4 (SAS Institute Inc., Cary, NC, US).

## Results

### Cohort characteristics

We identified 439,682 unique individuals from the 2014 NRD who were hospitalized with acute ischemic stroke between January 1, 2014 and November 30, 2014. Cohort sociodemogaphic, clinical, and care setting characteristics are provided in Table 1. Nearly all (95.1%) index AIS hospitalizations were emergency admissions, with AIS documented 82.7% of the time as the primary reason for hospitalization. Overall, 4.7% of AIS inpatients were identifed as having experienced one or more medication-related adverse events during their index hospitalization, with 98.0% of medication-related adverse events being recorded as secondary diagnoses (conditions unlikely responsible for occasioning the admission).

**Table 1:** Characteristics of index AIS hospitalizations

Characteristic	Index Events n (%) *n* = 439,682
Age	
< 40	12,878 (2.9)
40–49	25,172 (5.7)
50–59	64,364 (14.6)
60–69	95,136 (21.6)
70–79	103,821 (23.6)
80–89	101,941 (23.2)
90+	36,371 (8.3)
Sex	
Female	225,570 (51.3)
Male	214,111 (48.7)
Primary payer^a^	
Private insurance	75,983 (17.3)
Medicare	295,655 (67.2)
Medicaid	39,091 (8.9)
Self-pay	16,303 (3.7)
No charge	2308 (0.5)
Median household income	
$66,000+	88,803 (20.2)
$51,000 - $65,999	99,932 (22.7)
$40,000 - $50,999	118,495 (27.0)
$1 - $39,999	126,668 (28.8)
Missing	5783 (1.3)
Length of stay	
0–7 days	306,786 (69.8)
> 7 days	132,896 (30.2)
Discharge disposition	
Routine	174,933 (39.8)
Transfer: short-term hospital	8551 (1.9)
Transfer: other type of facility	164,884 (37.5)
Home health care	86,989 (19.8)
Against medical advice	3531 (0.8)
Discharged alive, destination unknown	794 (0.2)
Comorbidities	
0–2	110,756 (25.2)
3–4	166,434 (37.9)
5–6	107,199 (24.4)
7+	55,292 (12.6)
Bed size of hospital	
Small	64,949 (14.8)
Medium	120,363 (27.4)
Large	254,370 (57.9)
Control/ownership of hospital	
Government, non-federal (public)	53,708 (12.2)
Private, not-for-profit (voluntary)	326,024 (74.1)
Private, investor-owned (proprietary)	59,950 (13.6)
Teaching status of hospital	
Metropolitan teaching	278,770 (63.4)
Metropolitan non-teaching	118,548 (27.0)
Non-metropolitan	42,364 (9.6)

^a^Some categories excluded due to small sample sizes

### Readmissions

In total, 47,170 (10.7%) of hospitalized individuals with AIS were readmitted within 30 days of discharge. The rate of all-cause 30-day readmission for inpatients hospitalized with AIS who experienced medication-related adverse events (15.4%) was greater than that of AIS inpatients who did not experience such events (10.5%) (Table 2). Primary documented reasons for re-hospitalization were consistent with those observed among older adult populations and included cerebrovascular disease, septicemia, congestive heart failure, renal failure, and urinary tract infections.

**Table 2:** Primary reasons for readmission within 30 days of hospital discharge

All Index Encounters		Index Encounters With Medication-Related Adverse Event		Index Encounters Without Medication-Related Adverse Event	
*n* = 47,170 / 439,682; Readmission Rate = 10.7%	n (%)	*n* = 3200 / 20,822; Readmission Rate = 15.4%	n (%)	*n* = 43,970 / 418,860; Readmission Rate = 10.5%	n (%)
Acute cerebrovascular disease	5833 (12.4)	Septicemia	464 (14.5)	Acute cerebrovascular disease	5591 (12.7)
Septicemia	5197 (11.0)	Acute cerebrovascular disease	241 (7.5)	Septicemia	4733 (10.8)
Congestive heart failure; nonhypertensive	1974 (4.2)	Congestive heart failure; nonhypertensive	165 (5.1)	Congestive heart failure; nonhypertensive	1809 (4.1)
Acute and unspecified renal failure	1852 (3.9)	Acute and unspecified renal failure	131 (4.1)	Acute and unspecified renal failure	1720 (3.9)
Urinary tract infections	1462 (3.1)	Pneumonia	118 (3.7)	Urinary tract infections	1356 (3.1)
Cardiac dysrhythmias	1412 (3.0)	Urinary tract infections	106 (3.3)	Cardiac dysrhythmias	1321 (3.0)
Gastrointestinal hemorrhage	1329 (2.8)	Cardiac dysrhythmias	91 (2.8)	Gastrointestinal hemorrhage	1252 (2.8)
Pneumonia	1272 (2.7)	Respiratory failure; insufficiency; arrest	88 (2.7)	Late effects of cerebrovascular disease	1158 (2.6)
Aspiration pneumonitis; food/vomitus	1223 (2.6)	Complications of surgical procedures or medical care	86 (2.7)	Pneumonia	1154 (2.6)
Late effects of cerebrovascular disease	1193 (2.5)	Aspiration pneumonitis; food/vomitus	80 (2.5)	Aspiration pneumonitis; food/vomitus	1143 (2.6)

Of AIS inpatients readmitted within 30 days, 6205 (13.2%) were readmitted for AIS, with 40,965 (86.8%) readmitted for other reasons. Among individuals readmitted for AIS, those with a medication-related adverse event documented during their index hospitalization were more likely than those without to be readmitted between 2.4 and 15.0 days of discharge (*p* = 0.003) (Table 3). Time to readmission by medication-related event status during index hospitalization did not significantly differ for other examined reasons of readmission.

**Table 3:** Time to readmission by medication-related adverse event status during index hospitalization

Medication-related adverse event	Time to Readmission Median (IQR)	Time to Readmission Quartiles				*p* value^a^
		Quartile 1 n (%)	Quartile 2 n (%)	Quartile 3 n (%)	Quartile 4 n (%)	
	Any Readmission	1.0–4.0 days	4.1–10.0 days	10.1–18.6 days	18.7–30.0 days	
No	10.0 (4.0–18.5)	10,961 (24.9)	11,050 (25.1)	10,364 (23.6)	11,595 (26.4)	0.492
Yes	10.4 (4.5–18.8)	734 (22.9)	815 (25.5)	767 (24.0)	883 (27.6)	
	AIS Readmission	1.0–2.3 days	2.4–6.8 days	6.9–15.0 days	15.1–30.0 days	
No	6.9 (2.2–15.1)	1396 (23.5)	1329 (22.4)	1720 (28.9)	1499 (25.2)	0.003
Yes	6.7 (3.4–13.5)	31 (11.8)	83 (31.8)	89 (34.1)	58 (22.3)	
	Non-AIS Readmission	1.0–4.4 days	4.5–10.5 days	10.6–18.9 days	19.0–30.0 days	
No	10.5 (4.4–18.9)	8805 (23.2)	9547 (25.1)	9238 (24.3)	10,436 (27.4)	0.819
Yes	10.7 (4.7–19.0)	651 (22.1)	731 (24.9)	718 (24.5)	837 (28.5)	

Abbreviations: *AIS* Acute ischemic stroke, *IQR* Interquartile range

^a^Chi-square test

### Factors associated with readmission

Compared to AIS inpatients who did not experience medication-related adverse events, inpatients who experienced medication-related adverse events had significantly greater odds of being readmitted within 30 days (AOR, 1.22; 95% CI: 1.14–1.30) (Table 4). Medication-related adverse events were found to be significantly associated with acute readmission for non-AIS conditions (AOR, 1.26; 95% CI: 1.17–1.35); however, were not associated with readmission for AIS (AOR, 0.91; 95% CI: 0.75–1.10).

**Table 4:** Association between medication-related adverse events and 30-day readmission in AIS

Medication-related adverse event	30-Day Readmission		*p* value^a^	AOR^b^(95% CI)
	Yes n (%)	No n (%)		
	Any Readmission			
No	43,970 (93.2)	374,890 (95.5)	<.001	Reference
Yes	3200 (6.8)	17,622 (4.5)		1.22 (1.14–1.30)***
	AIS Readmission			
No	5944 (95.8)	412,916 (95.3)	0.172	Reference
Yes	261 (4.2)	20,561 (4.7)		0.91 (0.75–1.10)
	Non-AIS Readmission			
No	38,026 (92.8)	380,834 (95.5)	<.001	Reference
Yes	2939 (7.2)	17,883 (4.5)		1.26 (1.17–1.35)***

Abbreviations: *AIS* Acute ischemic stroke, *AOR* Adjusted odds ratio, *CI* Confidence interval

***p < 0.001

^a^Chi-square test

^b^Adjusted for age, sex, primary payer, number of Elixhauser comorbidities, median household income, length of stay, discharge disposition, bed size of hospital, hospital control/ownership, and hospital teaching status

Several other factors were associated with all-cause 30-day readmission (Table 5), including but not limited to: being younger than 40 years (AOR, 1.12; 95% CI: 1.00–1.26), Medicare (AOR, 1.33; 95% CI: 1.26–1.40) or Medicaid insurance (AOR, 1.41; 95% CI: 1.32–1.51) coverage, lowest income quartile (AOR, 1.08; 95% CI: 1.03–1.14), length of stay greater than 1 week (AOR, 1.38; 95% CI: 1.33–1.42), leaving hospital against medical advice (AOR, 2.41; 95% CI: 2.08–2.79), having 7 or more distinct comorbidites (AOR, 2.20; 95% CI: 2.08–2.34), and admission to a large hospital (AOR, 1.08; 95% CI: 1.02–1.15) or for-profit hospital (AOR, 1.20; 95% CI: 1.12–1.29) (Table 5). Individuals who were 90 or more years of age (AOR, 0.82; 95% CI: 0.74–0.91) and those who received care at non-metropolitan hospitals (AOR, 0.79; 95% CI: 0.73–0.85) were less likely to be readmitted following AIS hospitalization. Similar associations were observed for non-AIS readmissions (Table 5).

**Table 5:** Odds of readmission in AIS according to demographic, clinical, and care setting characteristics

Characteristic	Any Readmission		*p* value^a^	AOR^b^(95% CI)	AIS Readmission		*p* value^a^	AOR^b^(95% CI)	Non-AIS Readmission		*p* value^a^	AOR^b^(95% CI)
	Yes n (%) *n* = 47,170	No n (%)*n* = 392,512			Yes n (%)*n* = 6205	No n (%)*n* = 433,477			Yes n (%)*n* = 40,965	No n (%)*n* = 398,717		
Age												
< 40	1311 (2.8)	11,567 (2.9)	<.001	1.12 (1.00–1.26)*	135 (2.2)	12,743 (2.9)	<.001	0.76 (0.55–1.04)	1176 (2.9)	11,702 (2.9)	<.001	1.20 (1.06–1.36)**
40–49	2379 (5.0)	22,793 (5.8)		Reference	365 (5.9)	24,807 (5.7)		Reference	2014 (4.9)	23,158 (5.8)		Reference
50–59	6212 (13.2)	58,151 (14.8)		0.97 (0.88–1.05)	929 (15.0)	63,434 (14.6)		1.01 (0.83–1.21)	5283 (12.9)	59,080 (14.8)		0.96 (0.87–1.05)
60–69	9649 (20.5)	85,487 (21.8)		0.93 (0.85–1.01)	1431 (23.1)	93,705(21.6)		1.08 (0.90–1.30)	8218 (20.1)	86,918 (21.8)		0.90 (0.83–0.99)*
70–79	11,992 (25.4)	91,829 (23.4)		0.97 (0.88–1.06)	1590 (25.6)	102,231 (23.6)		1.12 (0.91–1.37)	10,402 (25.4)	93,419 (23.4)		0.95 (0.86–1.05)
80–89	11,861 (25.1)	90,080 (22.9)		0.93 (0.85–1.02)	1389 (22.4)	100,551 (23.2)		0.99 (0.81–1.22)	10,472 (25.6)	91,469 (22.9)		0.93 (0.84–1.02)
90+	3766 (8.0)	32,605 (8.3)		0.82 (0.74–0.91)***	366 (5.9)	36,005(8.3)		0.72 (0.56–0.93)*	3400 (8.3)	32,971 (8.3)		0.84 (0.75–0.94)**
Sex												
Female	24,509 (52.0)	201,061 (51.2)	0.087	Reference	3024 (48.7)	222,546 (51.3)	0.013	Reference	21,485 (52.4)	204,085 (51.2)	0.005	Reference
Male	22,661 (48.0)	191,450 (48.8)		1.03 (1.00–1.07)	3181 (51.3)	210,930 (48.7)		1.07 (0.99–1.17)	19,480 (47.6)	194,631 (48.8)		1.02 (0.99–1.06)
Primary payer^c^												
Private insurance	5834 (12.4)	70,149 (17.9)	<.001	Reference	1002 (16.1)	74,981 (17.3)	0.159	Reference	4832 (11.8)	71,151 (17.8)	<.001	Reference
Medicare	34,216 (72.5)	261,439 (66.6)		1.33 (1.26–1.40)***	4160 (67.0)	291,495 (67.2)		1.02 (0.90–1.16)	30,057 (73.4)	265,598 (66.6)		1.39 (1.30–1.47)***
Medicaid	4794 (10.2)	34,297 (8.7)		1.41 (1.32–1.51)***	636 (10.2)	38,455 (8.9)		1.20 (1.02–1.42)*	4158 (10.2)	34,933 (8.8)		1.44 (1.34–1.55)***
Self-pay	1264 (2.7)	15,040 (3.8)		1.04 (0.93–1.16)	231 (3.7)	16,072 (3.7)		1.05 (0.81–1.37)	1033 (2.5)	15,270 (3.8)		1.03 (0.92–1.16)
No charge	169 (0.4)	2139 (0.5)		1.01 (0.72–1.41)	42 (0.7)	2266 (0.5)		1.38 (0.85–2.25)	127 (0.3)	2181 (0.5)		0.92 (0.64–1.34)
Median household income												
$66,000+	9260 (19.6)	79,543 (20.3)	<.001	Reference	1312 (21.1)	87,492 (20.2)	0.014	Reference	7948 (19.4)	80,855 (20.3)	<.001	Reference
$51,000 - $65,999	10,175 (21.6)	89,756 (22.9)		0.97 (0.92–1.02)	1327 (21.4)	98,605 (22.7)		0.90 (0.79–1.03)	8848 (21.6)	91,083 (22.8)		0.99 (0.94–1.04)
$40,000 - $50,999	12,546 (26.6)	105,950 (27.0)		1.01 (0.96–1.06)	1544 (24.9)	116,952 (27.0)		0.88 (0.78–1.01)	11,002 (26.9)	107,493 (27.0)		1.04 (0.98–1.09)
$1 - $39,999	14,620 (31.0)	112,048 (28.5)		1.08 (1.03–1.14)**	1952 (31.5)	124,717 (28.8)		1.03 (0.91–1.17)	12,668 (30.9)	114,000 (28.6)		1.09 (1.03–1.15)**
Missing	569 (1.2)	5214 (1.3)		n/a	71 (1.1)	5712 (1.3)		n/a	498 (1.2)	5285 (1.3)		n/a
Length of stay												
0–7 days	27,070 (57.4)	279,716 (71.3)	<.001	Reference	4557 (73.4)	302,229 (69.7)	<.001	Reference	22,513 (55.0)	284,273 (71.3)	<.001	Reference
> 7 days	20,100 (42.6)	112,796 (28.7)		1.38 (1.33–1.43)***	1648 (26.6)	131,248 (30.3)		0.75 (0.68–0.83)***	18,452 (45.0)	114,444 (28.7)		1.50 (1.44–1.55)***
Discharge disposition												
Routine	13,069 (27.7)	161,864 (41.2)	<.001	Reference	2265 (36.5)	172,667 (39.8)	<.001	Reference	10,803 (26.4)	164,129 (41.2)	<.001	Reference
Transfer: short-term hospital	1306 (2.8)	7245 (1.8)		1.91 (1.70–2.14)***	327 (5.3)	8224 (1.9)		3.08 (2.44–3.90)***	979 (2.4)	7571 (1.9)		1.63 (1.44–1.84)***
Transfer: other type of facility	22,491 (47.7)	142,394 (36.3)		1.52 (1.45–1.59)***	2168 (34.9)	162,716 (37.5)		1.06 (0.95–1.19)	20,322 (49.6)	144,562 (36.3)		1.59 (1.51–1.67)***
Home health care	9693 (20.5)	77,296 (19.7)		1.26 (1.20–1.32)***	1298 (20.9)	85,691 (19.8)		1.19 (1.07–1.33)**	8395 (20.5)	78,594 (19.7)		1.27 (1.20–1.34)***
Against medical advice	595 (1.3)	2936 (0.7)		2.41 (2.08–2.79)***	##	##		3.12 (2.29–4.23)***	450 (1.1)	3081 (0.8)		2.13 (1.83–2.47)***
Discharged alive, destination unknown	16 (0.0)	777 (0.2)		0.20 (0.11–0.38)***	##	##		0.16 (0.02–1.08)	15 (0.0)	779 (0.2)		0.21 (0.11–0.42)***
Comorbidities												
0–2	7125 (15.1)	103,632 (26.4)	<.001	Reference	1364 (22.0)	109,392 (25.2)	0.001	Reference	5761 (14.1)	104,995 (26.3)	<.001	Reference
3–4	16,020 (34.0)	150,414 (38.3)		1.36 (1.30–1.43)***	2400 (38.7)	164,034 (37.8)		1.20 (1.07–1.34)**	13,620 (33.2)	152,814 (38.3)		1.40 (1.32–1.47)***
5–6	14,413 (30.6)	92,786 (23.6)		1.78 (1.68–1.87)***	1629 (26.2)	105,571 (24.4)		1.31 (1.16–1.47)***	12,784 (31.2)	94,415 (23.7)		1.86 (1.75–1.97)***
7+	9612 (20.4)	45,680 (11.6)		2.20 (2.08–2.34)***	812 (13.1)	54,479 (12.6)		1.34 (1.15–1.55)***	8800 (21.5)	46,492 (11.7)		2.34 (2.19–2.49)***
Bed size of hospital												
Small	6334 (13.4)	58,615 (14.9)	<.001	Reference	920 (14.8)	64,028 (14.8)	0.533	Reference	5414 (13.2)	59,535 (14.9)	<.001	Reference
Medium	12,747 (27.0)	107,616 (27.4)		1.04 (0.98–1.11)	1762 (28.4)	118,601 (27.4)		1.02 (0.88–1.17)	10,985 (26.8)	109,379 (27.4)		1.05 (0.97–1.12)
Large	28,089 (59.5)	226,281 (57.6)		1.08 (1.02–1.15)*	3523 (56.8)	250,847 (57.9)		1.00 (0.88–1.13)	24,566 (60.0)	229,803 (57.6)		1.10 (1.02–1.18)*
Control/ownership of hospital												
Government, non-federal (public)	5348 (11.3)	48,360 (12.3)	<.001	Reference	732 (11.8)	52,976 (12.2)	0.081	Reference	4616 (11.3)	49,093 (12.3)	<.001	Reference
Private, not-for-profit (voluntary)	34,592 (73.3)	291,432 (74.2)		1.04 (0.97–1.11)	4529 (73.0)	321,495 (74.2)		1.00 (0.86–1.16)	30,063 (73.4)	295,961 (74.2)		1.04 (0.97–1.12)
Private, investor-owned (proprietary)	7229 (15.3)	52,720 (13.4)		1.20 (1.12–1.29)***	944 (15.2)	59,006 (13.6)		1.09 (0.91–1.30)	6286 (15.3)	53,664 (13.5)		1.21 (1.13–1.30)***
Teaching status of hospital												
Metropolitan teaching	30,821 (65.3)	247,949 (63.2)	<.001	Reference	3856 (62.1)	274,914 (63.4)	0.161	Reference	26,965 (65.8)	251,806 (63.2)	<.001	Reference
Metropolitan non-teaching	12,662 (26.8)	105,886 (27.0)		0.97 (0.93–1.01)	1786 (28.8)	116,762 (26.9)		1.08 (0.97–1.19)	10,876 (26.5)	107,671 (27.0)		0.95 (0.91–1.00)*
Non-metropolitan	3687 (7.8)	38,677 (9.9)		0.79 (0.73–0.85)***	563 (9.1)	41,801 (9.6)		0.92 (0.77–1.11)	3124 (7.6)	39,240 (9.8)		0.77 (0.71–0.84)***

##Data suppressed - 10 or fewer observations in some cells

Abbreviations: *AIS* Acute ischemic stroke, *AOR* Adjusted odds ratio, *CI* Confidence interval

***p < 0.001; **p < 0.01; *p < 0.05

^a^Chi-square test

^b^Adjusted for all factors listed in table

^c^Some categories excluded due to small sample sizes

Having Medicaid insurance (AOR, 1.20; 95% CI: 1.02–1.42), leaving the hospital against medical advice (AOR, 3.12; 95% CI: 2.29–4.23), and having 7 or more distinct comorbidites (AOR, 1.34; 95% CI: 1.15–1.55) were identified as some of the factors associated with readmission for AIS. Conversely, individuals who were 90 or more years of age (AOR, 0.72; 95% CI: 0.56–0.93) and those who were hospitalized for more than 1 week (AOR, 0.75; 95% CI: 0.68–0.83) were less likely to be readmitted for AIS following their initial AIS hospitalization.

## Discussion

We analyzed nationally representative administrative claims from 439,682 individuals hospitalized with AIS between January and November 2014 in the US in order to characterize reasons for acute readmission and determine whether inpatient medication-related adverse events and select characteristics independently predicted readmission. Primary findings indicated that 10.7% of individuals were readmitted within 30 days of AIS discharge, that readmissions were primarily for reasons frequently observed in older adult populations, and that experiencing a medication-related adverse event greatly increased the likelihood of being readmitted within 30 days for reasons other than AIS. Our secondary findings showed that: (1) several factors, including, but not limited to, younger age, public health insurance, leaving the hospital against medical advice, and multimorbidity, were associated with an increased odds of all-cause acute readmission, and (2) that the eldest individuals and those receiving care at non-metropolitan hospitals were less likely to be readmitted following inpatient care for AIS.

Dramatic reductions in stroke incidence and related mortality have been observed in the US during the last decade and the age-adjusted AIS hospitalization rate decreased by 18.4% between 2000 and 2010 [2], with improvements being similar across sex and race [3]. Nevertheless, stroke remains a leading national cause of hospitalization and patient disability. Readmission following AIS hospitalization is common, with a large study of Medicare beneficiaries hospitalized for AIS in 2005–2006 reporting that 14.4% of patients were readmitted within 30 days of discharge and that nearly 12% of all readmission were for preventable causes [4]. During the year following AIS discharge, rates of readmission are estimated to be as high as 27% [14]. Our finding that 10.7% of patients with AIS were readmitted within 30 days of discharge suggests that there has been little overall improvement to the national rate of post-stroke acute readmission in recent years. Acute readmissions following AIS hospitalization in the US are almost always (~ 90%) unplanned, are associated with more than $17 billion of annual healthcare spending, and in some circumstances may serve as an indicator of the quality of healthcare services received [5]. Although not all unplanned readmissions are preventable, knowledge of modifiable and non-modifiable risk factors associated with readmission provides the opportunity to develop validated readmission risk prediction tools to identify patients at greatest risk of acute readmission. Ideally, such tools could help target the delivery of health services, including enhanced discharge planning, to those found to be at great risk of readmission. In turn, this may improve individual health outcomes and lead to more efficient healthcare spending [26].

Medication-related adverse events may include side effects to medications taken as prescribed, accidental overdosing by patients, and medication errors [22]. Such events contribute to acute readmission and up to 50% of medication-related adverse events occurring post-discharge are considered preventable [27, 28]. Recently, a study of more than 500 readmissions to a large academic hospital in the US found that approximately 13% of all 30-day preventable readmissions were attributable to medication-related adverse events [27]. Of readmissions classified as preventable, one half were the result of prescribing errors, while the other half resulted from insufficient patient monitoring or education [27]. These findings highlight potential population and health system benefits that may result from interventions aimed at mitigating the risk of experiencing medication-related adverse events.

To date, studies of risk factors for readmission following inpatient stroke care have largely focused on patient or stroke-related factors, including, but not limited to, age, sex, socioeconomic status, place of residence, medical history, and the National Institutes of Health Stroke Scale/Score [4–6, 17]. Similar to our findings, prior investigations have consistently identified recurrent stroke, infections, and cardiac conditions as primary causes of acute readmission in AIS, and longer length of index AIS hospitalization and increasing morbidity to be independently associated with readmission [4, 17, 29]. Other reported patient and stroke-related risk factors for short- and long-term readmission after stroke include prior coronary heart disease, heart failure, and diabetes, as well as having a feeding tube or urinary catheter [17]. Although we have confirmed prior reports that patient and geographic disparities in AIS exist and contribute to observed undesirable health outcomes such as acute readmission, characteristics such as individual age, sex, socioeconomic status, and place of residence are unlikely to be modified. At best, knowledge of these particular disparities may provide baseline data for future population-based research into other patient-level risk factors for readmission after AIS.

The path from patient and geographic disparity to timely, effective, and sustainable intervention is presumed to be very long and uncertain. In consideration of these challenges, our study focused on the association between medication-related adverse events in the inpatient setting and acute readmission after AIS discharge, since medication-related adverse events are potentially modifiable events that may affect all patients. Our research adds to the growing body of literature pertaining to the role of hospital environments, both physical and social, in contributing to readmission after hospitalization for AIS [14, 17, 30, 31]. Specifically, our finding that medication-related adverse events during hospitalization for AIS are associated with acute readmission may in part explain readmission risks observed among patients with AIS treated by hospitalists (compared to non-hospitalists; hazard ratio, 1.30; 95% CI: 1.11–1.52) [14] and those receiving care at hospitals with higher use of hospice (compared to non-use; OR, 5.86; 95% CI: 1.13–30.3) [30]. Moreover, our findings provide a clinically important contribution to knowledge of potentially avoidable outcomes related to modifiable events occurring within the hospital environment, and inspire new questions pertaining to inpatient medication monitoring and drug safety, areas that are often understudied in neurology.

Our study has a number of strengths. We used nationally representative and longitudinal health claims data and a US-validated algorithm to identify inpatients with AIS [20]. As such, it is unlikely that individuals included in our study were admitted primarily for hemorrhagic stroke, transient ischemic attack, traumatic brain injury, or other cerebrovascular conditions. Inpatient medication-related adverse events were coded using a method reported by the Agency for Healthcare Research and Quality to be efficient at classifying drug-related events in HCUP data [22]. In-depth demographic, clinical, and hospital NRD data enabled us to examine the association between various potential risk factors and acute readmission, and to control for several likely sources of confounding in our analyses. Collectively, our study methods permitted an assessment of the national inpatient burden of AIS with high external validity.

Despite these strengths, our study has certain limitations. Individuals receiving AIS care at hospitals outside of their home state were excluded from our study since it is not possible to track longitudinal follow-up across state boundaries. Therefore, the reported number of individuals admitted to hospital during our study period likely underestimates the true burden of AIS. It is also possible that not all medication-related adverse events experienced by inpatients were adequately documented within the source administrative claims data for this study. Notwithstanding this potential bias, misclassification of medication-related adverse event status is presumed to be non-differential and would therefore bias our reported associations towards the null. In addition, it remains possible that extraneous variables that could not be controlled for within our study (such as stroke severity, outpatient medication use, and comorbidities diagnosed prior to index hospitalization) may confound reported associations between examined risk factors and readmission.

## Conclusions

In conclusion, using nationally representative health claims data from the US, we found that readmission within 30 days of inpatient care for AIS is common and associated with medication-related adverse events occurring during the index hospitalization. Although study replication is warranted, our initial findings provide compelling evidence that acute readmissions in AIS may be avoidable through implementation of interventions focused on modifiable risk factors within the hospital environment. Future studies are needed to fully elucidate how potentially modifiable factors within the hospital environment (such as medication monitoring activities, pharmacist involvement in patient management, available nursing expertise, and communication methods) influence patient risk of medication-related adverse events and acute readmission. Population-based studies are also required to characterize inpatient medication-related adverse events in AIS by medication class and determine whether such adverse events and associated readmissions are avoidable. Lastly, future studies should examine whether the odds of acute readmission after AIS hospitalization vary by type of medication-related adverse event experienced during the index admission.

## Abbreviations

AIS: Acute ischemic stroke
AOR: Adjusted odds ratio
CI: Confidence interval
HCUP: Healthcare Cost and Utilization Project
ICD-9: International Classification of Diseases, Ninth Revision
IQR: Interquartile range
NRD: Nationwide Readmissions Database
US: United States

## References

[1] Mozaffarian D, Benjamin EJ, Go AS, Arnett DK, Blaha MJ, Cushman M, Das SR, de Ferranti S, Despres JP, Fullerton HJ (2016). Executive summary: heart disease and stroke statistics--2016 update: a report from the American Heart Association. Circulation.

[2] Ramirez L, Kim-Tenser MA, Sanossian N, Cen S, Wen G, He S, Mack WJ, Towfighi A (2016). Trends in acute ischemic stroke hospitalizations in the United States. J Am Heart Assoc.

[3] Koton S, Schneider AL, Rosamond WD, Shahar E, Sang Y, Gottesman RF, Coresh J (2014). Stroke incidence and mortality trends in US communities, 1987 to 2011. JAMA.

[4] Lichtman JH, Leifheit-Limson EC, Jones SB, Wang Y, Goldstein LB (2013). Preventable readmissions within 30 days of ischemic stroke among Medicare beneficiaries. Stroke.

[5] Bhattacharya P, Khanal D, Madhavan R, Chaturvedi S (2011). Why do ischemic stroke and transient ischemic attack patients get readmitted?. J Neurol Sci.

[6] Nahab F, Takesaka J, Mailyan E, Judd L, Culler S, Webb A, Frankel M, Choi D, Helmers S (2012). Avoidable 30-day readmissions among patients with stroke and other cerebrovascular disease. Neurohospitalist.

[7] Chin DL, Bang H, Manickam RN, Romano PS (2016). Rethinking thirty-day hospital readmissions: shorter intervals might be better indicators of quality of care. Health Aff (Millwood).

[8] Lichtman JH, Leifheit-Limson EC, Jones SB, Watanabe E, Bernheim SM, Phipps MS, Bhat KR, Savage SV, Goldstein LB (2010). Predictors of hospital readmission after stroke: a systematic review. Stroke.

[9] Hansen LO, Young RS, Hinami K, Leung A, Williams MV (2011). Interventions to reduce 30-day rehospitalization: a systematic review. Ann Intern Med.

[10] Centers for Medicare & Medicaid Services (CMS) Readmissions Reduction Program. https://www.cms.gov/Medicare/Medicare-Fee-for-Service-Payment/AcuteInpatientPPS/Readmissions-Reduction-Program.html. Accessed 10 Dec 2017.

[11] Chen C, Scheffler G, Chandra A (2014). Readmission penalties and health insurance expansions: a dispatch from Massachusetts. J Hosp Med.

[12] Fonarow GC, Smith EE, Reeves MJ, Pan W, Olson D, Hernandez AF, Peterson ED, Schwamm LH (2011). Hospital-level variation in mortality and rehospitalization for medicare beneficiaries with acute ischemic stroke. Stroke.

[13] Hsieh CY, Lin HJ, Hu YH, Sung SF (2017). Stroke severity may predict causes of readmission within one year in patients with first ischemic stroke event. J Neurol Sci.

[14] Howrey BT, Kuo YF, Goodwin JS (2011). Association of care by hospitalists on discharge destination and 30-day outcomes after acute ischemic stroke. Med Care.

[15] Lichtman JH, Leifheit-Limson EC, Jones SB, Wang Y, Goldstein LB (2012). 30-day risk-standardized mortality and readmission rates after ischemic stroke in critical access hospitals. Stroke.

[16] Bjerkreim AT, Thomassen L, Brogger J, Waje-Andreassen U, Naess H (2015). Causes and predictors for hospital readmission after ischemic stroke. J Stroke Cerebrovasc Dis.

[17] Rao A, Barrow E, Vuik S, Darzi A, Aylin P (2016). Systematic review of hospital readmissions in stroke patients. Stroke Res Treat.

[18] Leitao A, Brito A, Pinho J, Alves JN, Costa R, Amorim JM, Ribeiro M, Pinho I, Ferreira C (2017). Predictors of hospital readmission 1 year after ischemic stroke. Intern Emerg Med.

[19] Kilkenny MF, Longworth M, Pollack M, Levi C, Cadilhac DA (2013). Factors associated with 28-day hospital readmission after stroke in Australia. Stroke.

[20] Tirschwell DL, Longstreth WT (2002). Validating administrative data in stroke research. Stroke.

[21] Quan H, Sundararajan V, Halfon P, Fong A, Burnand B, Luthi JC, Saunders LD, Beck CA, Feasby TE, Ghali WA (2005). Coding algorithms for defining comorbidities in ICD-9-CM and ICD-10 administrative data. Med Care.

[22] Lucado J, Paez K, Elixhauser A (2011). Medication-related adverse outcomes in U.S. hospitals and emergency departments, 2008: statistical brief #109. Healthcare cost and utilization project (HCUP) statistical briefs.

[23] U.S. Agency for Healthcare Research and Quality. HCUP Clinical Classifications Software (CCS). Healthcare Cost and Utilization Project (HCUP). Updated March 2017. http://www.hcup-us.ahrq.gov/toolssoftware/ccs/ccs.jsp. Accessed 10 Dec 2017.

[24] U.S. Agency for Healthcare Research and Quality. 2014 Introduction to the NRD. Healthcare Cost and Utilization Project (HCUP). www.hcup-us.ahrq.gov/db/nation/nrd/NRD_Introduction_2014.jsp. Accessed 10 Dec 2017.

[25] U.S. Agency for Healthcare Research and Quality. HCUP Methods Series: Calculation Nationwide Readmissions Database (NRD) Variances. Report # 2017–01. https://www.hcup-us.ahrq.gov/reports/methods/2017-01.pdf. Accessed 18 Dec 2017.

[26] Kansagara D, Englander H, Salanitro A, Kagen D, Theobald C, Freeman M, Kripalani S (2011). Risk prediction models for hospital readmission: a systematic review. JAMA.

[27] Dalleur O, Beeler PE, Schnipper JL, Donze J. 30-day potentially avoidable readmissions due to adverse drug events. J Patient Saf. 2017. 10.1097/PTS.0000000000000346. 10.1097/PTS.0000000000000346 28306610

[28] Davies EC, Green CF, Mottram DR, Rowe PH, Pirmohamed M (2010). Emergency re-admissions to hospital due to adverse drug reactions within 1 year of the index admission. Br J Clin Pharmacol.

[29] Fehnel CR, Lee Y, Wendell LC, Thompson BB, Potter NS, Mor V (2015). Post–acute care data for predicting readmission after ischemic stroke: a Nationwide cohort analysis using the minimum data set. J Am Heart Assoc.

[30] Burke JF, Skolarus LE, Adelman EE, Reeves MJ, Brown DL (2014). Influence of hospital-level practices on readmission after ischemic stroke. Neurology.

[31] Smith MA, Frytak JR, Liou JI, Finch MD (2005). Rehospitalization and survival for stroke patients in managed care and traditional Medicare plans. Med Care.

